# Microbiological aspects of microbial electrolysis cells: Isolation, screening, and electron transfer mechanisms

**DOI:** 10.3934/microbiol.2026019

**Published:** 2026-07-07

**Authors:** Twisampati Das, Bhawna Kandwal, Riddhi Chakraborty, Harsh Jadhav, Ayesha Mulla, Rajesh Kanawade, Suhas S. Shetye, Ibrahim M. Banat, Surekha K. Satpute

**Affiliations:** 1 Department of Microbiology, Savitribai Phule Pune University, Pune 411007, Maharashtra, India; 2 CSIR-National Chemical Laboratory, Dr. Homi Bhabha Road, Pune 411008, Maharashtra, India; 3 CSIR-National Institute of Oceanography, Dona Paula, 403004, Goa, India; 4 School of Biomedical Sciences, Ulster University, Coleraine, Northern Ireland, BT52 1SA, UK

**Keywords:** biohydrogen, exo-electrogen, biofilms, microbes, microbial electrolysis cells, substrates

## Abstract

Biohydrogen is a leading candidate in the global shift toward renewable energy, offering a sustainable solution to reduce reliance on fossil fuels. This review underscores the use of microbial electrolysis cells (MECs) as a viable strategy to address increasing energy demands. In MECs, extracellular electron transfer (EET) is a vital mechanism that enables microorganisms to channel electrons (e⁻) from organic donors to external acceptors. Exo-electrogenic bacteria, viz., *Geobacter*, *Shewanella*, *Pseudomonas*, *Nitrospira*, and *Desulfovibrio*, are frequently reported for their ability to utilize Iron: Fe (III) and Manganese: Mn (III/IV) as electron acceptors, which can be replaced with electrodes to drive proton reduction and hydrogen (H_2_) generation. The use of renewable substrates in fermentation processes further strengthens the commercial feasibility of biohydrogen production, while the molecular traits of microbes provide insights into optimizing H_2_ production. Despite encouraging laboratory-scale demonstrations, challenges persist due to variability in recovery methods and the limited microbial diversity explored. Expanding the repertoire of exo-electrogens and refining MEC configurations are critical to achieving higher yields. Overall, biohydrogen stands out as a clean, eco-friendly energy resource, with microbial electrolysis offering a sustainable energy technology.

## 1. Introduction

The dwindling supply of fossil fuels and other non-renewable energy sources is a growing global challenge. With the ever-increasing human population, the demand for energy, particularly renewable energy, continues to rise [1]. It is important to mention here that non-renewable energy sources are not infinite. These energy sources are being rapidly depleted by extensive global use across industries, leading to the release of CO_2_, CO, and other greenhouse gases, contributing to environmental pollution and requiring urgent scientific intervention [2],[3]. This necessitates a transition towards cleaner, renewable, and alternative energy sources (wind, solar, and hydropower) to address the aforesaid concerns. Among the possible existing solutions, hydrogen (H_2_) is gaining global attention as a direct energy carrier and a promising fuel for the future due to its high chemical energy potential [4]. Various H_2_ production methods i) thermal water electrolysis, ii) dark fermentation iii) gasification, iv) fossil fuel reforming, v) photoelectrochemical cells, vi) artificial photosynthesis, and vii) aqueous phase reforming, have been explored [4]–[6]. Environmental safety and economic feasibility, however, remain key challenges for widespread acceptance.

General methods (steam reforming, water electrolysis, gasification, and pyrolysis) employed for H_2_ production typically require different processing temperatures [7]. Moreover, H_2_ production from natural gas is relatively cost-effective but often accompanied by emissions of greenhouse gases. Based on the level of carbon emissions, four major terms, Brown, Grey, Blue, and Green, are popular for H_2_ production [8]. Brown H_2_ is produced from coal, which results in the emission of the highest CO_2_. Grey H_2_, derived from natural gas, also contributes remarkably to CO_2_ emissions. In contrast, Blue H_2_, generated through carbon capture and storage, results in considerably lower carbon emissions, whereas Green H_2_, produced using renewable energy for water electrolysis, emits virtually no CO_2_ [8],[9]. Table 1 presents some of the sources used in H_2_ production, along with the associated risk level and estimated carbon emissions.

**Table 1.** Utilization of different sources for the production of H_2_, along with emission of CO_2_ and levels of associated pollution risks.

Sources of H_2_ production	Level of the risk	Emission of carbon (kg CO_2_/kg H_2_)	Ref.
Coal	High risk	~37	[10]
Oil	High risk	~28	[11]
Natural gas	Moderate risk	~19	[12]
Nuclear energy	Low risk	~1.5	[13]
Small hydro	Low risk	~1.5	[14]
Geothermal	Low risk	~2.5	[15]
Biomass	Comparatively safer	0.0	[16],[17]

According to the International Energy Agency, IEA 2023, it is projected that by 2070, H_2_-based fuels will account for ≈ 500 million metric tons of the energy used in the transportation sector-shipping, aviation, and road transport. Among the forms, biohydrogen holds a promise as a sustainable fuel, as it produces zero carbon emissions and requires minimal electricity during its production compared to the conventional routes. Green H_2_ or biohydrogen production has emerged as a global focal point in the quest for justifiable energy solutions due to the increasing awareness of its potential and has since been rapidly gaining traction worldwide. Germany leads the green energy market. In May 2024, the German government allocated USD 746 million to ThyssenKrupp's Nucera project. The main focus of this project was ‘Electrolysis Technologies’, predominantly the production of green H_2_ at a commercial level. The project covered >600 electrolysis projects and more than 10 gigawatts of installed capacity worldwide. Collectively, the German state and the federal governments have committed ≈ USD 14.19 billion across 24 major projects aimed at enhancing H_2_ production infrastructure.

Japan is also advancing biohydrogen production. From Japan, the Fukushima H_2_ Energy Research Field (FH_2_R), one of the world's major H_2_ production facilities, utilizes solar energy for H_2_ generation [18]. Japan aims to scale up its green H_2_ output substantially in the near future, with the government planning to invest USD 98.8 billion between 2024 and 2029 under its Basic H_2_ Strategy. In the United States, the Department of Energy has announced funding of USD 750 million for 52 projects across 24 states to significantly lower the cost of H_2_ production while reinforcing leadership in the rapidly expanding H_2_ economy. Similarly, the French government has pledged USD 7.53 billion to support businesses involved in the green H_2_ sector. In India, the National Green H_2_ Mission was approved by the Union Cabinet on 4 January 2022, with a target to develop a green H_2_ production capacity of 5 million metric tons per annum by 2030. Key Indian players such as GAIL Pvt. Ltd, National Thermal Power Corporation (NTPC) Ltd., Adani New Industries Ltd., and Indian Oil Corporation are actively investing in biohydrogen production technology [19]. Additionally, academic institutions like the Bioprocess Engineering Laboratory at the Indian Institute of Technology, Kharagpur, and CSIR-National Chemical Laboratory, Pune, and Agharkar Research Institute, Pune, Maharashtra, are known for their work on biohydrogen production through technological routes [20].

To address the challenges associated with conventional H_2_ production, biological methods, particularly those involving microorganisms, have arisen as assured alternatives to produce biohydrogen. The use of renewable resources such as biomass further supports the development of sustainable biohydrogen production strategies while minimizing waste generation. The concept of harnessing energy from bacteria was originally introduced in 1911 by M. C. Potter [21], who demonstrated that bacteria could degrade organic substrates to release energy. One of the most innovative advancements in this domain is the Microbial Electrolysis Cells (MECs) technology, which represents a cutting-edge bio-electrochemical system that leverages microbial activity to generate H_2_ from organic waste, particularly in wastewater. Capitalizing on the unique metabolic processes of specific microorganisms, MECs favorably provide a low-energy alternative to traditional water electrolysis, requiring minimal external power input to drive H_2_ production.

Review articles [4],[6],[11],[16],[17],[21],[22] provide information on biohydrogen-production through MECs, detailing the reactor designs, exo-electrogens-producing microorganisms, electrode materials utilized, and the production level of H_2_. Nevertheless, some important aspects like diversity or microbial cultures reported for H_2_ production, screening methods/approaches available to identify potential microbial cultures, remain inadequately addressed.

This review underscores the microbiological aspects of MECs, where critical information on microbial isolation and screening of electroactive and H_2_-producing microorganisms, along with their roles in biofilm formation and extracellular electron (e⁻) transfer, have been collated. We examine the current state of MEC technology and outline the underlying mechanisms of extracellular e⁻ transfer in bacteria. Concise and informative insights into the most used bacterial genera in MEC technology, highlighting their H_2_-producing capabilities, are presented. Additionally, we discuss MEC reactor's designs, its components, and electrode materials employed and evaluate their effect on H_2_ production along with technological advancements, advantages, and bottlenecks. The use of renewable substrates in fermentation processes from a commercial perspective is detailed. This microbiology-focused perspective delivers insights into the biological aspects of MEC efficiency and sustainable production of biohydrogen. Thus, we aim to bridge the gap between fundamental electrochemistry, microbiology, and engineering solutions by systematically mapping the literature, establishing a roadmap of MECs technologies for researchers and industry professionals working towards sustainable development.

## 2. Methods

An extensive literature search was carried out using reliable scientific databases. Research articles, review papers, case studies, and reports published over the past two decades, with particular emphasis on the last five years, were analyzed. Advances, challenges, and future prospects of biohydrogen production through MECs are discussed. The figures were created using BioRender software (Licensed Professional Science Figure Creator, BioRender, Toronto, ON, Canada), while tables were prepared to facilitate the comparative analysis of the compiled data.

## 3. Overall Scenario of MEC Technology

Among the emerging technologies, the MEC has attracted considerable research interest due to its energy efficiency. In general, the MEC system has an anode and a cathode that are placed either within the same compartment or in separate chambers separated by a membrane that enables selective movements of protons (H^+^). The system operates with the application of a very low external voltage (0.2 and 1 V) [22]–[24], which facilitates e⁻ transfer between the electrodes. The optimal voltage required for efficient operation is lower than that required for conventional water electrolysis (1.23 V) [22]–[24]. Exo-electrogenic bacteria form biofilms over the anode surface and metabolize the supplied organic substrates, producing H^+^, e⁻, and CO_2_. The anode serves as the terminal e⁻ acceptor, while at the cathode, the e⁻ and H^+^ combine to generate molecular H_2_ [6],[25]. Both electrodes in an MEC system are submerged in a fermentation broth inoculated with the selected exo-electrogenic organisms responsible for H_2_ production. A bacterial growth medium and an external power source are used to facilitate e⁻ transfer from the anode toward the cathode [26]. The microbial communities metabolize the feedstock (in the fermentation broth) at the anode, releasing e⁻ and H^+^, while H_2_ gas is subsequently generated at the cathode. MEC technology was developed in the early 2000s to address challenges in wastewater treatment, particularly in the United States and the Netherlands [22],[23],[27]. In recent years, its application in H_2_ production has been progressively established. MEC is inherently an anaerobic process, wherein microbial communities, either as individual strains or in consortia, consume organic substrates or produce gases (CH_4_ or H_2_).

## 4. Reactor configurations in MEC systems

The optimization of MEC configuration aims to achieve high H_2_ yields while minimizing the cost of the reactor design. Various MEC designs/reactor configurations have been developed, considering factors such as the system's size, shape, inoculum type, an electrode spacing [28]. In this section, we describe the most common MEC configurations reported in the literature. Single-chamber MECs, double-chamber MECs, H-type cells, tubular configurations, and planar configurations are popular. ‘Single Chamber MECs’ comprises the placement of both the electrodes together in the same compartment without any membrane. The main advantage of this system is its very low level of internal resistance, and consequently, low voltage requirement for H_2_ production. Additionally, membrane biofouling issues are not found with the single-chamber MECs. Typically, a single-compartment or chamber MEC is less costly than a double-chamber configuration, as the latter is designed to reduce methanogenesis and produce a purer form of H_2_. Single-chamber MEC is better for CH_4_ production rather than pure H_2_ [29]. ‘Double-chamber MEC’ is where different anolytes and catholytes are used. The membrane placed between the electrodes reduces the substrate crossover between the chambers, consequently, improving biohydrogen production in the cathode chamber. However, the placement of membranes between the electrodes introduces internal resistance, which hinders e⁻ flow from the anode to the cathode [22],[23]. The dual-chamber designs are comparatively complicated and may pose complications in operation and while scaling up. The double-chamber system is generally applied at a laboratory-level setup where domestic wastewater is used for the production of biohydrogen [29],[30].

‘H-type MECs’ is a variation of double-chamber MEC that is generally applied at the laboratory level to examine the production of biohydrogen using organic waste [31]. The nomenclature ‘H-type’ denotes the shape, where two chambers (vertical position) are connected through a bridge (horizontal). This type of design separates the processes (biological and chemical) into two compartments (anode and cathode), divided by an ion-exchange membrane. At the anode chamber, exo-electrogens oxidize organic matter, generating e⁻ and H^+^. Further e^-^ travels through an external circuit under the influence of low voltage, whereas the H^+^ travels to the cathode chamber through the membrane and synthesizes H_2_ in comparatively purer form. The design of H-type MECs prevents methanogens (at the anode) from consuming the H_2_ produced. A major drawback with the H-type design is the possibility of having high internal resistance, which affects the yield of H_2_ sand, thus making it impractical for large-scale industry-level plants.

‘Tubular MEC configuration’ comprises two concentric tubular chambers, with the inner tube radially pierced and placed into a larger outer tube. An anode assembly is rolled in a circular manner around a central plastic rod within the inner tube, while a cation exchange membrane (CEM) is enclosed externally to cover the perforations, forming a partition between the two chambers. Furthermore, the cathode assembly is also wrapped around the CEM. Upon application of an external voltage, this design achieves a high H_2_ production rate, with a coulombic efficiency (CE) of 60%, H_2_ recovery of 45%, and an H_2_ yield of 1.1 mol per mol of acetate consumed. These results correspond to a 30.5% reduction in chemical oxygen demand (COD) [23]. In a subsequent study, a 1 L tubular MEC system achieved an H_2_ production rate of 7.1 L·H_2_·L^−1^·d^−1^. Furthermore, the tubular configuration maintains consistent internal resistance during scale-up, demonstrating its scalability and strong potential for large-scale biohydrogen production [32],[33]. A ‘Planar configuration’ makes use of flat and parallel electrodes stacked in close proximity to compress the internal dead volume. This sandwich-like structure maximizes spatial efficiency, reduces internal resistance, and distributes the electric field uniformly across the biofilm [34].

A key distinction between MEC and other H_2_ production technologies, like water electrolysis, lies in its use of electroactive marine bacteria (*Geobacter* spp. and *Shewanella* spp.), which facilitate e⁻ transfer [35]. Figure 1 represents a simplified schematic of a dual-chamber MEC system biohydrogen production housing the two electrodes, divided by an ion exchange membrane, and does not encompass all possible MEC biochemical and electrochemical pathways.

**Figure 1.**
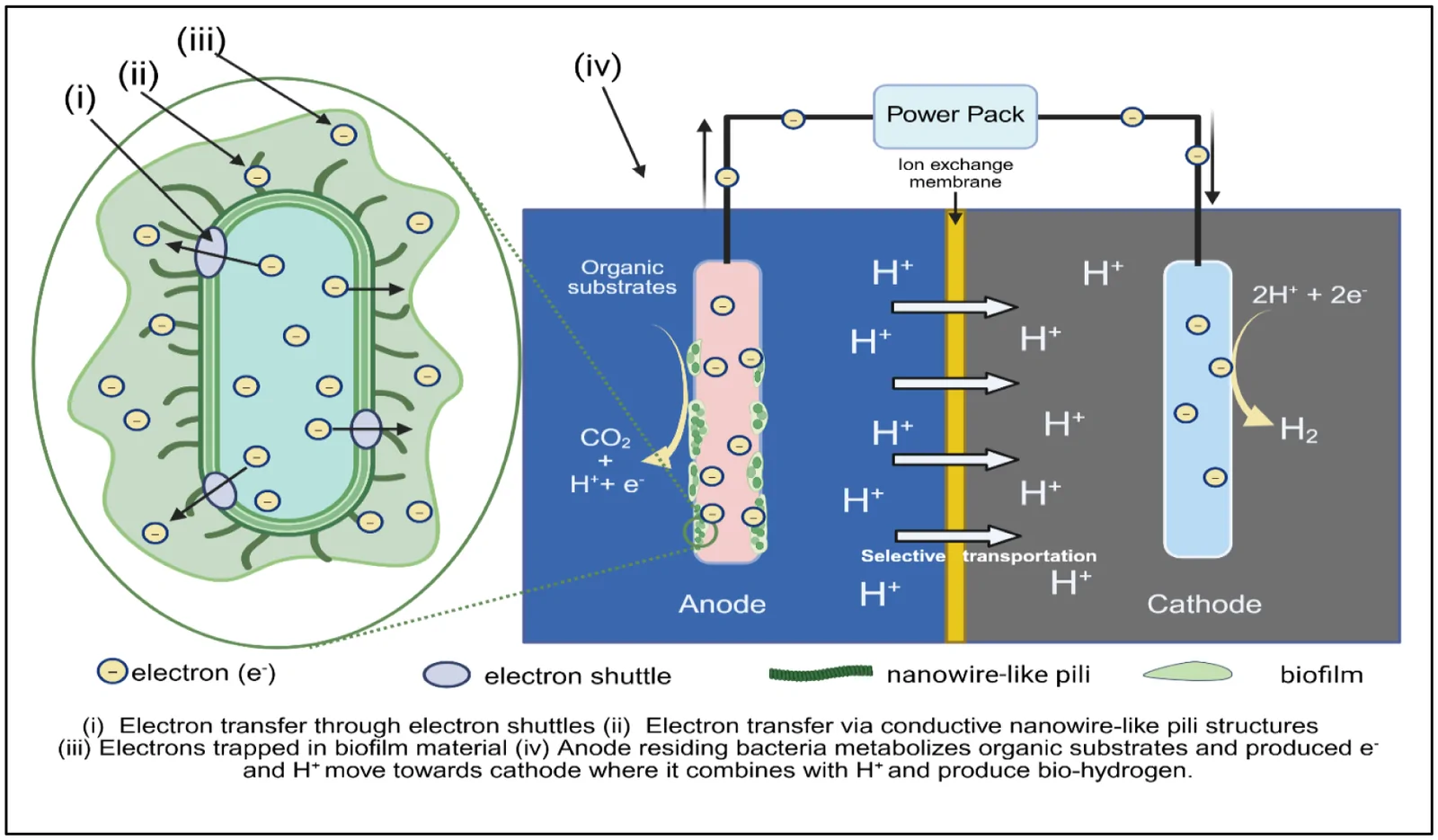
Schematic representation of a two-chamber (blue and grey) MEC system comprising two electrodes, separated by an ion-exchange membrane (yellow), illustrating biofilm-mediated electrochemical processes for biohydrogen production.

### 4.1. Electrode materials and their effect on H_2_ production in MEC technology

#### 4.1.1. Anode material

The decisive aim of MEC technology is to maximize H_2_ production to enable its industrial-scale application. Four major components significantly influence MEC performance: 1. Electrode material, 2. Substrate, 3. Membrane system, and 4. Exo-electrogen producer, i.e., the microbial culture. The primary role of the anode is to support the adhesion and colonization of H_2_-producing bacteria in the MEC setup. It facilitates the acceptance of e⁻ from microbial metabolism and their subsequent transfer to the cathode. The efficiency of e⁻ transfer largely depends on the functional moieties that exist on the anode surface [36]. Developing biofilms on the anode is a critical step, as enables greater bacterial attachment or adherence, which is influenced by the surface porosity and roughness of the anode [37],[38]. Since bacterial outer membranes generally carry a negative charge, they exhibit enhanced adhesion to positively charged anode surfaces. Thus, increasing the surface positive charge enhances bacterial attachment [39]. Commonly used anode materials are graphite, nickel foam, carbon felt, carbon mesh, carbon cloth, and carbon brushes [33]. Among these, nickel foam has demonstrated the highest efficiency as an anode material to date [40]. Each material varies in its conductivity, biocompatibility, surface properties, and cost, all of which are factors that collectively impact H_2_ yield in MEC systems. Table 2 provides a summary of widely used electrode materials as anode and their characteristics.

**Table 2.** Commonly used anode materials, their advantages, limitations, and H_2_ output.

Anode	Operating conditions	Advantages	Limitations	H_2_ yield L·H_2_·L^−1^·d^−1^	Ref.
Biochar-coated Ni foam (algal biochar)	Kitchen wastewater, Mixed algal biomass, 1–3 V supply, for 3 days. MEC-1, 2, 3, 4) configurations	Higher H_2_ yield, increased electroactive surface area Low-cost and energy-efficient MEC electrode	MEC-1 and MEC-2 did not perform well like MEC-3 and MEC-4	10.99	[41]
Graphitedialysis-bag	Artificial wastewater Dialysis bag anode, Bare anode, *Geobacter* medium	Enhanced electroactivity	Mechanical durability issues	0.076	[42]
Nickel foam (NF)	Single chamber MEC supplemented with acetate, Ni-P alloy coated Ni-Foam	High surface area for biofilm	Expensive, rigidity and oxide formations	2.29 ± 0.11	[43]
Graphite felt	Two-chamber MEC fed with acetate substrate, graphite felt, cathode, Inoculation with 10 mL *Desulfovibrio* sp., PO_4_^3−^ buffered medium (0.1 mL/L)	High conductivity	High cost	0.63	[44]
Carbon cloth	Sodium acetate/ cowshed wastewater Pre-acclimatized bioanodes immobilized with sodium alginate, anode treated with Pt	Better surface area	Low mechanical strength	0.39	[45]
Carbon felt	Slurry-farm soil, domestic wastewater, Anaerobic sludge, Cow dung, Fed-batch mode–13 days	High conductivity, more porosity	Expensive, potential for clogging	0.0069	[46]
Carbon mesh	Heating (450 °C/30 min) the carbon mesh in a muffle furnace result maximum power density (922 mW/m^2^, 46 W/m^3^)	Cheaper than carbon cloth, less clogging	Pre-treatment is necessary	1015 mW m^−2^*	[47]
Granular Graphite (GG)	Electrode-Filler for bioanode (< 4 mm). Mixed synthetic carbonaceous solution – substrate, pH 9/25 °C	Methanogenesis inhibition (pH > 9), High energy efficiency	High energy consumption, Internal biofilm resistance	1.28	[48]

Note: MES: Microbial electrolysis cell; NF: Nickel foam; MEC-1: Single pristine NF anode and single pristine NF cathode; MEC-2: Single pristine biochar-coated NF anode and pristine NF cathode; MEC-3: Four pristine NF anodes and four pristine NF cathodes; MEC-4: Four pristine biochar-coated NF anodes and four pristine NF cathodes; Pt: Platinum; GG: Granular graphite; *H_2_ production is correlated to the power density generated by the reactor.

#### 4.1.2. Cathode material

Catalysts play a decisive role in accelerating H_2_ production by improving reaction kinetics and reducing overpotential. Rapid catalysis can be achieved by selecting appropriate cathode materials, which influence e⁻ transfer efficiency [49]. In many MEC systems, the same material may be employed for the anode and cathode, with the cathode typically requiring an additional catalyst. Metals such as platinum (Pt) and palladium (Pd) are widely used as catalysts at the cathode, owing to their superior catalytic activity [33],[50]. However, nickel (Ni) serves as a cost-effective alternative, offering high catalytic efficiency at a significantly lower price [40]. Table 3 outlines some of the popular materials that are being used as cathode electrodes and their respective properties.

**Table 3.** Commonly used cathode materials, their advantages, limitations, and output of H_2_.

Cathode	Operating conditions	Advantages	Limitations	H_2_ yield L·H_2_·L^−1^·d^−1^	Ref.
FeS-coated carbon cloth	Anaerobic sludge deposition of FeS-NPs on carbon cloth, Single chamber MEC (250 mL)	High energy efficiency, high-rate H_2_ generation	Saturation of current uptake, Risk-Toxic byproducts, Long-term stability issues	1.96 ± 0.09	[51]
CoMo/SS	Synthetic nutrient medium, Microbes-Anaerobic sludge, CoMo alloy catalyst deposited on pre-treated SS mesh	Improved H_2_ evolution performance, excellent stability	Scale-up	1.12	[52]
NiMo Ht	Catalyst alloy grown on selected metal matrix via electrodeposition. Complex synthetic nutrient broth (Sodium acetate, glucose). Dual chamber MEC	Higher performance than electrochemical synthesis, huge current densities, H_2_ production rates	High cost	81 ± 3	[53]
Platinum-coated carbon cloth	Mango pulp wastewater and PTFE treated electrode	High surface area, retain biomass	High cost	0.67	[54]
CoP-NF	Hydrothermal synthesis followed by Phosphidation, Dual chamber (100 mL), 1 g/L acetate in 50 mM PBS	Superior electro-chemical surface area, efficient, stable, and low-cost	Surface oxidation susceptibility	222 ± 20.3	[55]
Ni_2_P/C-coated on SS mesh	Electrode fabrication - slurry casting technique: 5% Nafion®-binding agent, Dual chamber (56 mL), Fed-batch mode	Chemical stability	Flat, less surface area	0.29	[56]
Nickel foam	Anaerobic sludge + diluted wastewater (5 L), Electrode surface area of 5400 m^2^m^−3^, pH 7, 1 V	High productivity, greater surface area	Quick decrease in MEC performance	19.07 ± 0.46	[57]

Note: Ni_2_P: Highly active nickel phosphide; FeS: Iron-sulphide; CoMo/SS: Cobalt–molybdenum (Co-Mo) alloy catalyst, SS: Stainless steel; NiMo Ht: Nickel molybdenum Hydrothermal; CoP-NF: Phosphating cobalt nickel foam; PTTE: Polytetrafluoroethylene; NPs: Nanoparticles; An appropriate combination of cathode materials and associated catalysts is essential for maximizing H_2_ yield.

In MEC, hydrogenases are the central enzymatic catalysts responsible for the H_2_ evolution reaction (HER) at the cathode [58]–[60]. These metalloenzymes are categorized by their active-site metal composition, with [FeFe]-hydrogenases offering exceptionally high turnover frequencies (up to 10,000 s^−1^) but remaining highly sensitive to oxygen (O_2_) [58],[59],[61]. Conversely, [NiFe]-hydrogenases are often preferred for biocathodes due to their superior O_2_ tolerance, enabling stable operation under less stringent anaerobic conditions [59],[61]. The catalytic mechanism involves H^+^ binding to the enzyme's bimetallic active site (such as the H-cluster in [FeFe] types) to form hydride intermediates, which then react with additional H^+^ to release molecular H_2_ [58],[59],[62].

Non-precious metal catalysts like Ni serve as cost-effective and robust alternatives to expensive Pt [63],[64]. Ni-based gas diffusion cathodes have higher stability (performance >45 days) while directly exposed to liquids (at the anode) in membraneless MECs. Under typical MEC conditions, specifically near-neutral pH, Ni often outperforms Pt, which can be inhibited by the salts and metals present in the medium [63]. Furthermore, the high porosity of Ni-modified cathodes facilitates rapid H_2_ transport, which minimizes the availability of H_2_ to hydrogenophilic methanogens, thereby limiting the loss of energy to unwanted CH_4_ production. Iron Sulfide (FeS) is another significant non-precious catalyst that enhances MEC performance by mimicking biological ferredoxin complexes. H_2_-producing microbes naturally utilize Fe-S within their cell membranes to catalyze HER; thus, FeS nanoparticles (NPs) on the cathode surface act as a “conductive bridge” that facilitates direct extracellular electron transfer (EET) between the electrode and the biofilm. FeS-modified biocathodes that have higher current uptake and denser microbial populations compared to pristine carbon electrodes, achieves record-high H_2_ yields of 7.01 mol H_2_/ mol glucose [51].

The overall efficiency of the MEC system can be further optimized by regulating iron additives and electrical stimulation modes. Supplementation with magnetite (Fe_3_O_4_) NPs has been shown to upregulate the expression of essential genes, such as the [FeFe]-hydrogenase gene (*Cei 1773*) and the [NiFe]-hydrogenase gene (*hucL*), up to 30-fold [60]. Maintaining an optimal iron concentration (approximately 1.0 mg/L) in the medium improves biocompatibility by reducing reactive oxygen species (ROS) and lowering e⁻ transfer resistance [65]. Additionally, employing intermittent electrical stimulation can alleviate the inhibitory effects of continuous fields, selectively enriching electroactive bacteria like *Desulfovibrio* and increasing the relative abundance of [NiFe] hydrogenase groups to promote efficient H_2_ utilization [66].

#### 4.1.3. Separating a membrane placed between the electrodes

Membranes are primarily used to separate the two electrodes in the MEC system and ensure the production of high-purity H_2_. The use of membranes also enables the independent selection of different catholytes and anolytes, enhancing flexibility in system design [67]. The presence of reducing bacteria, however, can lead to CH_4_ formation, which decreases the quality and quantity of H_2_ production [68]. Commonly used membranes include Cation Exchange Membranes (CEMs), which facilitate the selective transfer of ions such as Na⁺, K⁺, and H^+^. Because they transfer H^+^, these membranes are often referred to as H^+^ exchange membranes—PEM [67],[69]. While CEMs offer advantages, they also present some limitations. For instance, Nafion® membranes, a widely used type of CEM, can reduce permeability and lead to challenges in large-scale MEC production systems due to their high cost and performance constraints [70],[71]. Thus, there is a pressing need, to standardize membrane components that would offer high efficiency, potency, and cost-effectiveness for large-scale production.

Anion exchange membrane (AEM) and PEM are used in MECs for the optimization of pure H_2_ production. AEM is charged with positively charged ions (such as quaternary ammonium) that specifically enable the transport of anions (Cl⁻ and HCO_3_⁻). AEMs have demonstrated less internal resistance and less accumulation of H^+^ in anode chambers [72]. PEM is typically composed of fluorinated polymers like Nafion® membrane PEMs that are charged with negatively charged sulfonic acid groups and selectively transport H^+^ from anode to cathode [73]. Moreover, while PEMs are enabled in high mechanical stability and H^+^ conductivity, they also pose limitations like high material costs and transfer of other cations (Na^+^, K^+)^ present in wastewater [72]. Alternative polymeric/composite membranes have been proposed to overcome the limitations associated with AEM and PEM. Low-cost materials such as Sulfonated Polyether Ether Ketone (SPEEK), polybenzimidazole, porous poly-vinylidene fluoride (PVDF), and blended polymer-inorganic composites are used in research [72],[73]. These substances help in high chemical durability and reduce H_2_ gas crossover.

## 5. Substrates utilized for the production of biohydrogen

Substrates are one of the most important aspects in the MEC assembly. A wide range of organic substrates are being used to produce H_2_. The type of substrate significantly influences the performance of MEC, H_2_ yield, and growth pattern of exo-electrogens present on the anode surface of the system. Substrates can be categorized based on their complexity and the by-products formed from them.

### 5.1. Simple and well-defined substrates

These substrates are considered model candidates, as they are used primarily in basic research and optimization studies due to their exact known chemical composition. These features make it easier to investigate their thermodynamics and kinetic calculations of MEC systems. For example, acetate is one of the most widely used substrates in the MEC system, since almost all exo-electrogens efficiently utilize acetate to produce e⁻ and H^+^ [74]. Other substrates like lactate, propionate, butyrate, and ethanol are short-chain fatty acids and alcohols, which are often by-products in fermentation processes, behave as efficient e⁻ donors [75],[76]. The yield of H_2_ produced from glucose varies with respect to different substrates, as it is dependent on the extent of utilizing capability of the substrate by the organism. Microorganisms generally prefer substrates that yield specific intermediate metabolites according to their metabolic pathways and thermodynamic favorability. During glucose metabolism, many exoelectrogenic bacteria predominantly produce acetate and lactate as end products. These metabolites can serve as effective e⁻ donors for H_2_ production, thereby enhancing biohydrogen generation [77]. A study by Cui et al. [78] investigated glucose fermentation from food waste and H_2_ production using a single-chamber MEC under hyperalkaline conditions. H_2_ production was tested in the experiment using 1 g/L glucose at varied pH conditions (7, 9.5, and 11.2), applying voltages of 0.6, 1.2, and 1.6 V. At the alkaline pH of 11.2, under the highest current density (180 ± 9 A/m^2^), pure H_2_ (93.3 ± 1.2%) was produced with a yield of 7.72 ± 0.23 mol H_2_/mol glucose provided at 1.6 V. The reactor with a single chamber is found to be more efficient compared to the double chamber system with a membrane. Although an external power is required, the voltage needed is much lower than what is required for H_2_ production by water electrolysis. Singh et al. [79] demonstrated production of 5.9 L·H_2_·L^−1^·d^−1^ using a 10 L single-chamber at 1 V, thereby confirming the feasibility to scale the technology up for biohydrogen production. Varied substrate concentrations influence levels of biohydrogen production. Xue et al. [80] showed the role of different concentrations of food waste (FW). Around 9.14, 5.70, and 7.78 mL/gVS H_2_ production was reported at 10, 20, and 40 gVS/L of FW, respectively. In contrast, higher FW concentrations aided rapid and substantial acidification at the onset of fermentation, effectively suppressing the growth of non-H_2_-producing microbes and enabling a much greater H_2_ production (68.63–120.78 mL/gVS). FW is a cost-effective approach and can complement conventional substrates (glycerol, acetic acid, and lignocellulosic biomass). Some of the non-fermentable compounds, such as glycerol and acetic acid, work as a primary substrate due to their availability at a high product-to-substrate ratio [27],[81]. Acetic acid is a byproduct of glucose fermentation by yeast cultures, and instead of being discarded in sewage, it can be used for H_2_ production in MECs. Notably, H_2_ production is not limited to anaerobic or facultatively anaerobic microbes; aerobic bacteria such as *Alcaligenes eutrophus* have also been shown to produce H_2_ from formic acid [82].

### 5.2. Renewable biomass as a complex substrate

These substrates are crucial for the real-world application and commercial viability of MECs, as they link H_2_ production to waste treatment and resource recovery [83]. For example, wastewater biomass is usually cost-effective and readily available but varies in composition depending on its source. Wastewater produced from domestic sewage, municipal wastewater, livestock manure, landfills, food and beverage wastes are also used as substrates, as they are rich in energy and organic content [84],[85]. However, one of the major drawbacks is that they often require pre-treatment, which increases the cost of the overall production process. Agricultural wastes, such as cellulose, lignocellulosic agricultural residue [86],[87], and sugarcane bagasse, can also be good alternative substrates since they are abundant, sustainable, and cheap. Nevertheless, such substrates may also require additional treatments like hydrolysis for breaking down the complex substrate into simpler ones, since not all exo-electrogens can utilize complex substrates. The choice of substrate significantly influences the yield of H_2_ in MEC systems, as shown in Table 4.

**Table 4.** Effect of different substrates on the H_2_ production rate in MEC.

Substrate	Conc. (g/L)	MEC production volume (mL)	H_2_ Production rate (m^3^·H_2_·m^−3^·d^−1^)	Ref.
Sludge hydrolysate	−	250	30.57	[88],[89]
Glucose	2	250	1.96 ± 0.09	[51],[90]
*Sugarcane vinasse* (Fermented)	−	250	12.7	[91]
Ethanol	1.5	170	0.514	[92]–[94]
Industrial wastewater	0.386 ± 0.007	28	0.12 ± 0.02	[57],[95],[96]
Fermentation effluent	1	26	1.41	[97],[98]
Sugarcane bagasse	25	1500	3.5	[99]–[101]
Glucose (fermentable)	2	26	0.25 ± 0.03	[78],[102],[103]
Sodium acetate (non-fermentable)	2.72	40	50	[40],[104]
Synthetic-effluent lignocellulose (fermentable)	1	28	1.11 ± 0.13	[99],[105]
Domestic wastewater	0.204–0.481	584	0.154 (L H_2_·g^−1^)*	[95],[106]

*Note: H_2_ production based on chemical oxygen demand (COD) removal.

## 6. Microbiological aspects of MEC technology—Microbial communities, enrichment, isolation and screening approaches to detect exo-electrogen activity

Microorganisms are broadly classified into aerobic, anaerobic, facultative anaerobic, and microaerophilic groups based on their O_2_ requirements for functioning as a final e⁻ acceptor [107]. Exo-electrogens are a subset of microbes that typically thrive under anaerobic conditions and are capable of donating e⁻ extracellularly to external e⁻ acceptors [108]. The phenomenon of EET in such organisms primarily occurs through three mechanisms (Figure 1): (i) Via outer membrane cytochromes; (ii) through e⁻ shuttle molecules, often facilitated by biofilm formation on electrodes; and (iii) through conductive nanowire-like pili structures [108]–[112].

### 6.1. Microbial communities

The use of pure or mixed microbial cultures strongly affects biohydrogen production in the MEC system. When mixed microbial communities are used, there is always a chance of over-competition by methanogens over non-methanogens, hindering the production of H_2_ due to the increased production of CH_4_ and CO_2_ [113]. Although both single and dual chamber MEC are feasible, single chamber MEC is less preferred due to the loss of H_2_ gas via methanogenesis and microbial inhibition (intermediate of the electrolysis process). Dual-chamber MEC designs, which are separated by a PEM avoid crossover of O_2_, lead to improved yield of H_2_ (up to 10–30%) but compromise the simplicity of the MEC design [114].

The process of EET by microorganisms to transfer e⁻ to minerals in close vicinity to the cell surfaces [115],[116] is developed in two ways: Direct Electron transfer (DET) and Indirect Electron transfer (IET). In DET, e⁻ can move from the bacteria to electrodes, sovereign of external mediators, being in direct contact with insoluble substrates (iron or manganese minerals) [117]. This mechanism obliges microbes to be in close contact with outer e⁻ recipients and demands transfers through outer membrane proteins (c-type cytochromes) [118],[119]. The e⁻ transfer via the cytochrome pathway in *Geobacter sulfurreducens* has been studied using mutant strains with cytochrome c genes, which are either overexpressed or deleted [120]. For example, DET can be mediated by outer membrane c-type cytochromes in species like *G*. *sulfurreducens*, *Shewanella oneidensis*, and *Acidithio bacillus ferrooxidans* (e.g., OmcA-MtrCAB protein complexes) [121]. Not all microbes exhibit DET; instead, some use conductive membrane protrusions called nanowires, resembling bacterial pili. These structures facilitate e⁻ conduction, enabling cells to interact with distant electrodes or metal oxides and enable interspecies e⁻ transfer within biofilms [119]. For instance, sulfate-reducing bacteria (SRB) can generate current at the cathode in electrochemical conditions, which is more positive than the thermodynamic potential for cathode H_2_ production, thus exhibiting DET mechanism for uptake [122]. *G. sulfurreducens* transfer e⁻ via protein-based pili nanowires, while *S. oneidensis* MR-1 forms nanowires from outer-membrane and periplasmic extensions containing multiheme cytochromes for EET [123],[124].

In the case of IET, transfer of e⁻ occurs through small redox-active organic molecules, e⁻ shuttles, excreted by cells or added exogenously, that repeatedly cycle between reduced and oxidized states to substrates at some distance from the cell surface. This type of shuttle acts as an acceptor, transferring e⁻ to iron oxides before getting reduced, enabling microbes to interact with distant electrodes or metal oxides [115]. Thiols, phenazines, humic compounds, and quinones are prospective e⁻ shuttles, while different species secrete some extracellular carriers such as flavins and phenazine derivatives [125],[126]. Beblawy et al. [127] demonstrated the coexistence of DET and IET in *S*. *oneidensis*, which transfer e⁻ by direct contact with the e⁻ acceptor and produce flavins as extracellular e⁻ carriers simultaneously, like that in *Shewanella* [117]. Exogenous and endogenous are two categories of e⁻ shuttles, classified based on their presence in the surroundings or the inherent capability of the cell to synthesize them [118]. In *Geobacter*, e⁻ transfer relies mostly on extracellular proteins and nanowire extensions, with little evidence for soluble flavin participation [117]. Moreover, *Acetogens* also use the IET for H_2_ generation through soluble e⁻ shuttles [128]. In conclusion, DET occurs when microbes pass e⁻ directly to electrodes or minerals via outer‑membrane cytochromes or conductive nanowires, offering high efficiency but requiring close contact. In contrast, IET relies on soluble e⁻ shuttles such as flavins, quinones, or phenazines, enabling long‑range transfer but with lower efficiency due to diffusion losses. Evidence shows that while DET is dominant in model electroactive bacteria like *Geobacter* and *Shewanella*, IET is more widespread in natural communities because of mediator availability. Many microbes employ both mechanisms simultaneously, adapting to environmental conditions for optimal extracellular e⁻ transfer.

One of the most investigated electroactive bacteria, *G. sulfurreducens*, employs outer membrane cytochromes along with conductive pili (nanowires) to transfer e⁻ to the anode surface. *S*. *oneidensis* utilizes riboflavin as an e⁻ shuttle for extracellular e⁻ transfer. Beyond supporting respiration, the mechanisms of external e⁻ transfer also serve additional functions such as quorum sensing (QS). This remarkable communication system enables cells to regulate gene expression in response to their population density. It operates through the synthesis, release, and detection of autoinducers, i.e., signaling molecules. After the accumulation of autoinducer molecules up to a critical concentration, they initiate synchronized changes in gene expression, enabling the bacterial community to adapt collectively and coordinate behaviors such as biofilm formation, virulence, and utilization of several resources. For example, *Pelotomaculum thermopropionicum* has been shown to form conjugative pili that connects it with *Methanothermobacter thermautotrophicus*, facilitating interspecies e⁻ transfer [108].

Proteobacteria, one of the oldest and most diverse bacterial phyla, dominate around 40% of the exo-electrogenic communities within MEC systems. Among these, Gammaproteobacteria are the most prevalent, comprising about 62%, followed by Epsilonproteobacteria (19.3%) and Betaproteobacteria (17.6%) [129]. In addition to proteobacteria, members of the phylum Bacillota account for approximately 30%, while Bacteroidetes account for roughly 36% of the microbial population involved in MEC-driven biohydrogen production [44],[130]. Bioaugmentation with *Clostridium pasteurianum* revealed the feasibility of achieving a high yield of biohydrogen, which enhanced the system performance, resulting in an H_2_ yield of 3.07 mol H_2_/mol hexose. Moreover, bioaugmentation shifted the dominant metabolic pathway from butyrate to acetate, which is more favorable for H_2_ generation [77]. As a result, *C. pasteurianum* became the predominant species within the granules, accounting for 92.3% of the bacterial population [131]. Moreover, efficient and robust electroactive microbes from diverse ecological niches (marine, hot springs) have been explored (Table 5).

**Table 5.** Representative genera explored for H_2_ production and electrolytic activities in the MEC.

Bacterial genera	Requirement of O_2_	Electrogenic nature	H_2_ production	Ref.
*Shewanella putrefaciens*	Facultative anaerobic	Present	ND	[132]
*Aeromonas hydrophila*	Facultative anaerobic	Present	ND	[133]
*Desulfuromonas acetexigens*	Anaerobic	Present	ND	[134]
*Rhodopseudomonas palustris*	Anaerobic	Present	+	[135]
*Enterobacter cloaceae*	Facultative anaerobic	Present	+	[136]
*Klebsiella pneumoniae*	Facultative anaerobic	Present	+	[137],[138]
*Nitrospirota*	Anaerobic	ND	+	[139]
*Clostridium*	Anaerobic	ND	+	[140]
*Shewanella oneidensis*	Anaerobic	Present	+	[141]
*Bacillota* phylum	Anaerobic	Present	+	[142],[143]
*Geobacter sulfurreducens*	Anaerobic	Present	+	[144]
*Clostridium acetobutylicum*	Anaerobic	Present	+	[145]
*Desulfovibrio* sp.	Anaerobic	Present	+	[146]
*Pseudomonas*	Anaerobic	Present	+	[147]

Note: ND: Not documented; +: H_2_ production activity documented.

### 6.2. Enrichment of exo-electrogens/electroactive bacteria for biohydrogen production

Some bacteria mediate extracellular e⁻ transfer through naturally occurring humic acid in the soil, while others utilize self-produced compounds such as phenazines, flavins, and quinones [148]. Riboflavin and flavin mononucleotide in electroactive bacteria interact with cytochrome to form a semiquinone compound, which further accelerates the e⁻ transfer mechanism [149]. The applied potential difference enriches electroactive bacteria on the anode, with the positive charge attracting the e⁻ from the bacteria's natural microflora [150]. It has also been observed that the addition of specific substrates can further enrich these organisms. The well-known electroactive genera like *Geobacter* and *Shewanella* sp. showed activity in the presence of acetate and lactate as carbon sources [89],[104]. It has been reported that quorum sensing also helps with electrogenic activities of bacteria [151]. A study by Liu et al. [152] demonstrated that nitrous acid-treated activated sludge enhances the electroactivity of *Geobacter* sp. and *Pseudomonas* sp., which in turn stimulates H_2_ production in MEC systems. One of the vital factors influencing this process is the inclusion of anode material. Li et al. [153] reported that when Fe-S NPs are incorporated into the anode, it improves the attachment of exo-electrogens. Electrodes modified with hydrophilic groups (for example, −NH_2_, −COOH, −OH) also promote biofilm formation for *Desulfuromonas acetexigens* [154]. Further enrichment techniques should be developed to facilitate the discovery and isolation of other electroactive bacteria.

### 6.3. Microbial isolation and screening assays employed for the detection of exo-electrogen activity

Exo-electrogenic microbes, or extracellular e⁻-producing microbes, are found in various natural environments such as seawater, lakes, rivers, and extreme habitats like hot spring waters, eruption muds, and saline lakes. These microorganisms have evolved specialized biochemical and e⁻-donating/accepting pathways through the process of natural selection [155]. Therefore, a comprehensive understanding of microbial systems is crucial for isolating and identifying such organisms. Significant advances were made in isolating and detecting organisms exhibiting exo-electrogenic activity using several qualitative and quantitative assays to identify electroactive microbes. For instance, a study by Khandelwal et al. [156] documented the use of tungsten trioxide (WO_3_) nanomaterials in Luria-Bertani (LB) agar plates to detect electroactive bacterial growth, indicated by a color change in the medium. The researchers also employed an immuno-magnetic capture technique, using antibody-coated magnetic beads to bind and isolate electrogenic bacteria selectively. Barron et al. [157], on the other hand, developed a rapid, single-step isolation technique that uses biological laser printing with metal oxides, specifically titanium dioxide (TiO_2_), to selectively isolate electroactive microbes.

#### 6.3.1. Dye reduction-based electron transfer activity monitoring (DREAM) assay

Moreover, Aiyer et al. [158] developed a rapid and efficient qualitative method for detecting e⁻ transfer activity, termed the DREAM assay. In this method, methylene blue dye (50 µM) is added to actively growing microbial cultures, and the absorbance (at 660 nm) is measured at 10-second intervals over the course of one minute using a spectrophotometer. The reduction of the dye indicates the presence and e⁻ transfer activity of microbes. As a colorimetric test, it demonstrates good efficiency in detecting bacterial activity. The technique has some limitations, including uneven bacterial distribution in the culture broth, which may lead to false-negative results. Additionally, certain media components may reduce the dye, causing false-positive results.

#### 6.3.2. Manganese dioxide (MnO_2_) reduction assay

In 2022, Nazeer and Fernando [159] developed a chromogenic agar plating method for isolating exo-electrogens. This technique involved incorporating manganese dioxide (MnO_2_) into the agar medium, where the bacterial cultures under investigation were streaked or spread. After 48–72 hours of incubation, the initial black coloration of the MnO_2_-containing plates faded due to bacterial e⁻ transfer. The novel medium formulation is (g/L); glucose: 2, NH_4_Cl: 0.46, yeast extract: 0.1, peptone: 0.5, K_2_HPO_4_: 5.05, KH_2_PO_4_: 2.84, MnO_2_: 1.5, cysteine: 0.025, and agar: 15, with cysteine serving as an O_2_ scavenger. The development of a chromogenic agar medium for identifying exo-electrogens is a breakthrough in isolating such microbes. However, a few challenges hinder the technique's applicability, including a lower concentration of extracellular e⁻, which can lead to false-negative results. Moreover, not all microbes are capable of using Mn^4+^ as an e⁻ acceptor. Further research is therefore needed to improve such assays. The DREAM assay and MnO_2_ assays are illustrated in Figure 2 as representative assays and protocols used to identify electroactive microbes from marine samples.

In nature, multiple bacterial genera may grow synergistically in a common niche. Studies show that the most commonly found MEC bacteria are mutually dependent on each other to carry out different complex activities [160], and as a result, co-cultures or consortia are of interest. A consortium of *Enterobacter aerogenes*, a flavogenic *Bacillus* sp., *Pseudomonas* sp., and *Pichia kudriavzevii* was reported to enhance biofilm formation at the anode surface and increase bioelectricity production in MEC [161]. Another study showed that a co-culture of *Escherichia coli* and *P. aeruginosa* exhibited improved acetate utilization, subsequent biofilm formation, and electricity production [162]. Thus, the performance of the MEC system in terms of H_2_ production capacity, yield, rate, and process proficiency under varying physicochemical parameters is crucial [163].

**Figure 2.**
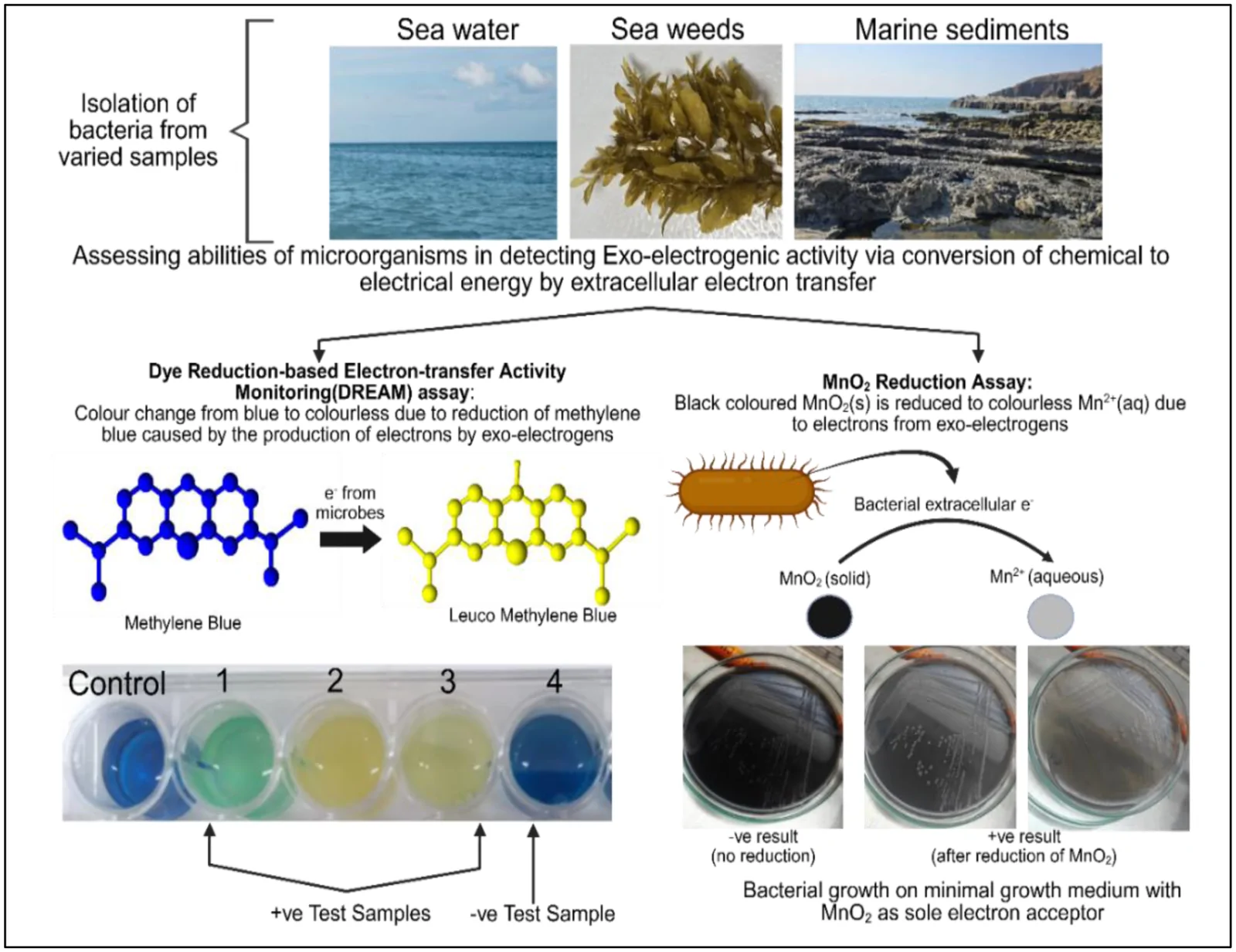
Representative assays employed for assessing exo-electrogen production abilities by microorganisms.

## 7. Limitations and potential improvement in MEC technology

The MEC technology has demonstrated effective performance at small laboratory scales; however, challenges arise when scaling up for industrial applications are attempted. In this section, we discuss the issues and challenges associated with current MEC operations.

### 7.1. Internal resistance and electrode spacing

Biohydrogen production through the MEC technology necessitates a reduction in internal resistance during the electrolysis process to maximize the current density and energy efficiency. The space between the electrodes affects the solution and the transfer resistance. Drastic reduction in the distance between the electrodes improves the generation of H_2_ through the MEC system only if the acceptable surface area is upheld. When the anode and cathode are placed closer to each other, it reduces the distance required for ions to travel through the solution, consequently lowering the internal resistance of the solution. It is important to differentiate the optimal and the minimal distance between the electrodes to minimize the resistance.

### 7.2. Energy efficiency and Coulombic efficiency

In the MEC system, energy efficiency represents the content of the total energy—biohydrogen generated against the energy inputs supplied before initiating the process. Two metrics, namely electrical input efficiency (*rW*) and total energy efficiency (*rE*), illustrate the efficiency of the MEC. The *rW* is >100% since it does not consider the free biochemical energy utilized from the substrates incorporated. *rE* considers not only external electricity but also the substrate enthalpy, where total energy efficiency is <100%, which can be attributed to two things within the cell: The inherent thermodynamic irreversible nature and internal ohmic resistances. To achieve the optimized metric, it is important to minimize hyper potential conditions at both the electrodes while working with very low voltages (approx. 0.2 to 0.8 V). This enables keeping the voltage input lower compared to the energy of the H_2_ gas produced. The CE denotes the fraction of e⁻ recovered in the form of electric current compared to the total e⁻ that was present through the organic substrates consumed. In MEC technology, the higher the CE means the biofilm forming exoelectrogenic microbial communities effectively direct e⁻ to the anode circuit instead of losing those entities through alternative metabolic pathways. Around 60 to 90% CE is observed to be dependent on the biomass produced, the fermentation reactions, and the competitive consumption of e⁻ by the microbial cultures used.

### 7.3. Reactor design constraints and capacity

Reactor configuration is another major aspect because industrial-scale MECs require containers larger than laboratory-scale ones. This requirement often results in greater distances between electrodes, resulting in reduced production efficiency. Several studies have shown that MECs can operate efficiently in volumes ranging from 1 to 10 L, yielding high amounts of H_2_ [79],[86]. Singh et al. [79] designed a 10 L MEC reactor where the electrode orientation manages to encourage mixing of fluid. The novel design of the MEC reactor is provided in such a way (electrode frames—metal sheet and copper wires) that it reduces the loss of energy. Researchers have demonstrated the performance of the reactor with small-scale MECs, where fermentation is carried out under optimal conditions using substrates that facilitate to 267.50 ± 0.50 mL/g of H_2_ production [86]. Nevertheless, reproducible laboratory results often fail to translate consistently at larger, industrial scales, creating obstacles for practical application [34],[164]. One of the major problems hindering large-scale MEC operations is CH_4_ contamination. Methanogens tend to form colonies on the anode, leading to a reduction in H_2_ output. To address this issue, a proton-exchange membrane has been introduced between the electrodes, resulting in the production of pure H_2_. However, the addition of the membrane has also led to a decrease in the overall H_2_ yield. A simple reactor design helps maintain a favorable surface-to-volume ratio, making operations easier. From an industrial perspective, a single-chambered reactor is particularly advantageous [165]. However, the cost of biohydrogen production remains a significant barrier, being nearly ten times higher than that of alkaline water electrolysis (AWE). This is one of the commercially used approaches to produce green H_2_. It splits a water molecule into H_2_ and O_2_ with the help of an external high electrical supply under alkaline conditions. Moreover, biohydrogen production from MEC is microbe-mediated through metabolic pathways by utilizing organic substrates [20]. In economic terms, AWE is an intensive process heavily dependent on electricity. The feedstock cost for AWE is significantly low due to the minimal requirement of water and KOH. The primary economic expenditure for AWE is the electricity (50–55 kWh) that is required to produce 1 kg of H_2_. On the contrary, the feedstock cost for MEC is free/negative as it utilizes wastewater as an organic substrate, which may reduce the cost and initiate the process toward green economy, and the electricity requirement is very low, around 1–2 kWh/kg of H_2_. The expenditure for MEC is dependent on fermenter designs, maintenance of strict anaerobic conditions, and inclusion of expensive metals (Pt). Modern designs are shifting towards better alternatives, such as Ni and carbon cloth/mesh anodes, to minimize the capital expenditure.

Several strategies have been developed to control methanogen growth in larger feedstock volumes, such as maintaining a low pH, limiting carbonate availability, and applying UV-IR irradiation, all of which have shown success in reducing the growth of methanogens [33],[34],[163]. Electrohydrogenesis in MECs enables H_2_ production at yields surpassing fermentation and at higher energy efficiencies than water electrolysis. Even though a membrane was once considered essential to prevent H_2_ gas losses, Call and Logan [166] demonstrated that single-chamber MECs without membranes achieved high H_2_ recoveries at the cathode (78 ± 1% to 96 ± 1%), having a voltage supply of 0.3–0.8 V (7.5 mS/cm solution conductivity). Energy efficiency relative to electrical input declined with increasing voltage, ranging from 406% at 0.3 V to 194% at 0.8 V, with overall efficiencies averaging 78%. Overall, maximum H_2_ production reached 1.02 ± 0.05 L·H_2_·L^−1^·d^−1^ at 0.4 V. Hence, these findings highlight that membrane-free MECs can sustain high H_2_ recovery and production rates, offering simpler and more cost-effective designs for industrial application [167]. Moreover, a dual-chamber model evades contamination of cathode metal catalysts from various microbial species. The main drawback of the dual-chamber model is that the membrane imposes higher internal resistance (up to 86%), thus limiting overall H_2_ production efficiency. In contrast, the single-chamber MECs eliminate membrane-related losses, enabling higher current densities. Additional benefits include simpler fabrication, reduced fouling, and improved operational ease, making them more suitable for scalable applications [168].

### 7.4. Comparison with pilot-scale and industrial-scale studies

Scaling up H_2_ production from laboratory-scale (< 1 L) to pilot-scale (from 10 to >1,000 L) by MECs intensely affects the overall efficiency due to the thermodynamic aspects and operational restrictions. It is easier to work at a laboratory level with lower volumes, where acetate-based synthetic and conductive substrates are utilized for the fermentation process. The close placement of electrodes results in the reduction of internal resistance compared to the industrial-level large-scale setups. At the industrial level, comparatively complex and unstable wastewater bodies impose challenges vastly different from those faced at the laboratory level. Overall, a shift from the laboratory level to the industrial scale calls for a huge transition for parameters (voltage inputs, current density, internal resistance, etc.). Moreover, large-scale operations are associated with mass-transfer challenges (due to large volumes) and a greater variation in pH that affects the biofilm-forming electrogenic microbial communities. Under such circumstances, there is a possibility of overgrowth of methanogenic archaeal genera over the preferred exo-electrogens. These methanogens may consume biohydrogen produced by the exo-electrogens and produce CH_4_, thus lowering the yield and purity of H_2_. To mitigate the H_2_ production losses at the larger scale, instead of a single larger size chamber, a stacked configuration may be considered, enabling attaining a greater surface-area-to-volume ratio of the electrodes. Nevertheless, extensive optimization is required for a pragmatic operation of reactors with volumes of at least 1000 L. This includes identifying a suitable and consistent feedstock for continuous biohydrogen production and selecting the ideal combination of electrodes to enable efficient scaling from laboratory set-ups to industrial reactors. Researchers should also prioritize cost-optimization strategies to overcome these challenges. Figure 3 illustrates the major challenges and their respective potential solutions aimed at enhancing the productivity of biohydrogen through the MEC approach.

**Figure 3.**
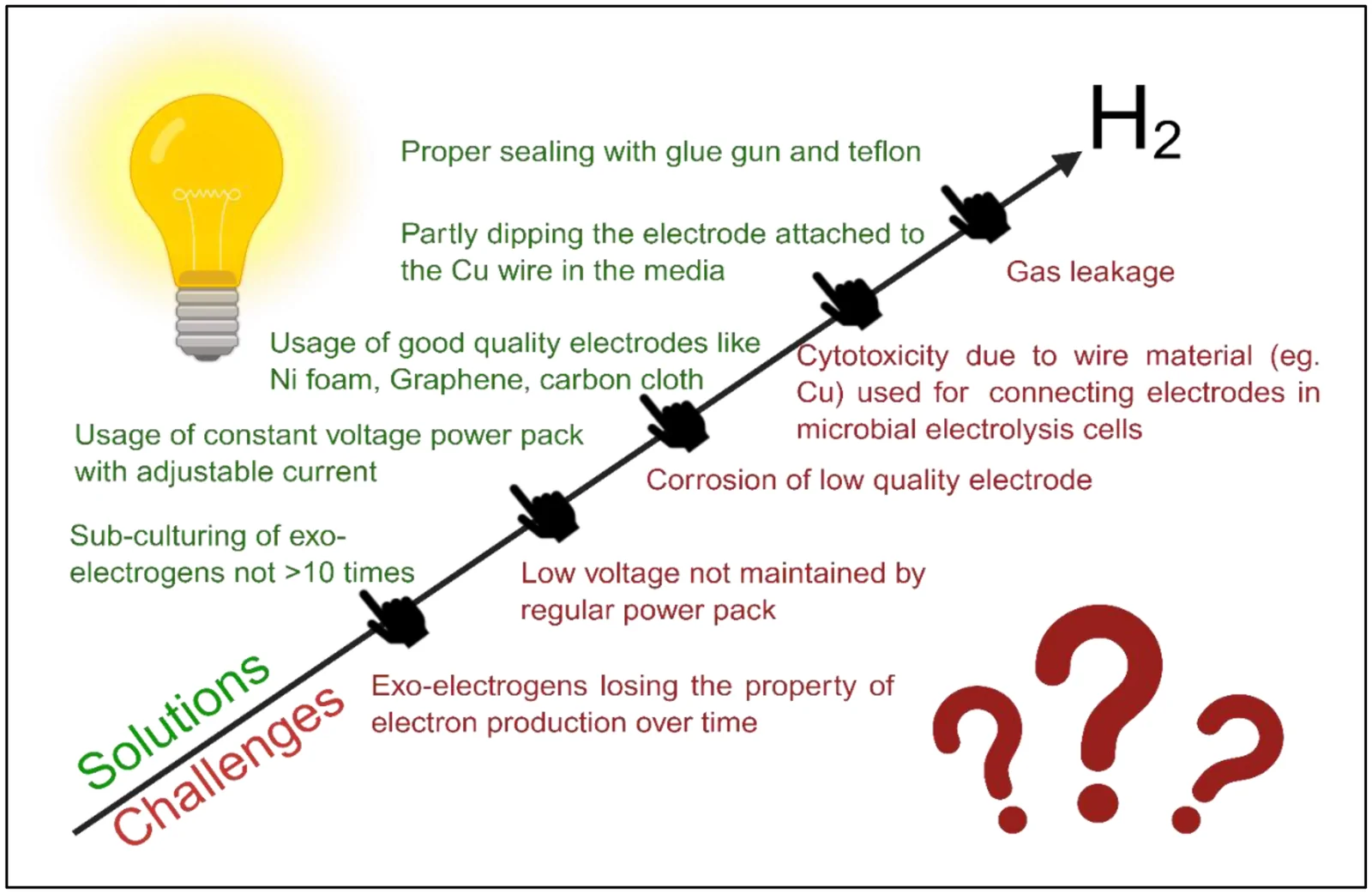
Suggested solutions to the challenges encountered during the production of biohydrogen through MECs.

## 8. Future prospects

The MEC technology is a promising platform designed to address the challenges associated with the depletion of non-renewable energy sources, offering a sustainable, zero-carbon emission, and environmentally friendly fuel alternative. The efficiency of biohydrogen production largely depends on the initial substrates used to cultivate exo-electrogenic microorganisms. The availability of cost-effective substrates is critical for the practical implementation of large-scale H_2_ production through MECs. Activated sludge and hypersaline marine sediments serve as effective substrates, given their microbial diversity and potential to reveal novel exo-electrogens. Furthermore, analysis of the microbial diversity and subsequent exo-electrogens screening are necessary requirements for advancing this field. The formation of microbial biofilms at anode surfaces are considered essential for efficient e⁻ transfer where potentially pathogenic microorganisms must be avoided.

Microbial consortia are promising in biohydrogen production and may hold significant practical potential to increase the yield. Extensive research is needed to optimize the biofilm formation (at anode), which involves selecting suitable electrode materials and identifying ideal microbe-electrode combinations, since not all electrodes are universally compatible with different microbial strains. Furthermore, system parameters (pH, temperature, substrate/s, and electrode composition) must be carefully standardized to improve MEC performance and obtain consistent biohydrogen yields. Despite the advancements in technical and economic aspects, challenges remain for future MEC technology scaling up processes from the laboratory to a commercial scale.

## 9. Conclusions

The MEC technology is remarkable in utilizing organic waste for the sustainable production of H_2_. Even though this technology has manifested noteworthy potential for sustainable biohydrogen production, many challenges hinder large-scale applications. Major limitations include: 1. Low H_2_ production rates under real wastewater conditions; 2. High costs of electrodes, electrode materials, their surface properties, and catalysts; 3. Membrane fouling; and 4. Competition from methanogenic microorganisms that reduce H_2_ recovery. Furthermore, most studies remain confined to laboratory-scale reactors, creating a substantial gap between experimental performance and commercial feasibility. Contradictory findings regarding optimal reactor configurations, microbial consortia, and substrate selection highlight the need for standardized methodologies, long-term pilot-scale studies, and comprehensive techno-economic assessments. Exo-electrogens such as *Geobacter* and *Shewanella* transfer e⁻ extracellularly via cytochromes, e⁻ shuttles, or conductive pili. Single-chambered designs are generally less expensive (due to low maintenance and high operational efficiency) compared to double-chambered designs. Moreover, waste biomass is a sustainable substrate for H_2_ production, aiding in waste valorization. Strategies, like modification of anode material, selective enrichment enhancing electroactive biofilm formation, and UV-IR irradiation that help control methanogen growth, are needed for reactors exceeding 1000 L. Isolation techniques, followed by screening through DREAM and MnO_2_-based assays, facilitate rapid detection of electroactive microbes. Sustainable feedstocks and effective electrode combinations are critical for a successful scale-up. Ultimately, cost reduction and technical improvements are essential to advance MEC technology towards successful industrial biohydrogen production. Continued innovations in system design, material optimization, active shifting toward membraneless reactor configurations, cost-effective nanomaterial electrodes, genetic engineering to optimize e⁻ transfer pathways, and microbial enrichment are vital for the industrial-scale. Effectively integrating these advancements through hybrid systems will be crucial to reducing production costs and establishing MECs as a financially viable technology within the circular bioeconomy.

## Use of AI tools declaration

The authors declare they have not used AI tool to generate, analyze, or interpret the scientific content. However, AI tool was partly used to improve the language and grammar of the article.
